# Assessment of Recombinant A2-Latex Agglutination Test (RA2-LAT) and RA2-ELISA for Detection of Canine Visceral Leishmaniasis: A Comparative Field Study with Direct Agglutination Test in Northwestern Iran

**Published:** 2018

**Authors:** Mahin FARAHMAND, Hossein NAHREVANIAN, Vahid KHALAJ, Mehdi MOHEBALI, Mohammad BARATI, Sanaz NADERI, Zabih ZAREI, Ghader KHALILI

**Affiliations:** 1. Dept. of Parasitology, Pasteur Institute of Iran, Tehran, Iran; 2. Dept. of Biotechnology, Pasteur Institute of Iran, Tehran, Iran; 3. Dept. of Medical Parasitology and Mycology, School of Public Health, Tehran University of Medical Sciences, Tehran, Iran; 4. Center for Research of Endemic Parasites of Iran (CREPI), Tehran University of Medical Sciences, Tehran, Iran; 5. Infectious Diseases Research Center, AJA University of Medical Sciences, Tehran, Iran; 6. Islamic Azad University, Science and Research Branch of Kurdistan, Sanandaj, Iran; 7. Meshkin-Shahr Research Station, School of Public Health, Tehran University of Medical Sciences, Meshkin-Shahr, Iran; 8. Dept. of Immunology, Pasteur Institute of Iran, Tehran, Iran

**Keywords:** Recombinant A2, Latex agglutination test, Canine visceral leishmaniasis, ELISA, Direct agglutination test, Iran

## Abstract

**Background:**

This study aimed to set-up latex agglutination test (LAT) and ELISA based on recombinant A2 from Iranian strain of *Leishmania (L.) infantum* (rA2-Ag) and evaluated for detection of anti-*Leishmania* antibodies in dogs compared to standard direct agglutination test (DAT).

**Methods:**

The rA2-Ag was synthesized under a part of the A2 gene sequences which contain immune dominant sequences and less number of repetitive sequences. Latex beads, 0.8 μm (Sigma, USA) were sensitized with rA2-Ag. The tests were carried out on sera collected from 350 ownership dogs including symptomatic (n=67), asymptomatic (n=230) canine visceral leishmaniasis (CVL), and (n=53) uninfected domestic dogs as control group.

**Results:**

Anti-leishmanial antibodies were detected in 97 (27.7%), 96 (27.4%) and 29 (%9) of the serum samples by using DAT, rA2-ELISA, and rA2-latex, respectively with ≥1:320 as a cut-off titer when DAT-confirmed cases were compared with the control groups. A combined sensitivity of 52% and specificity of 82.40% for rA2-ELISA and 23.8% and specificity 95.38%, respectively were found with ≥1:320 as a cut-off titer when DAT-confirmed cases were compared with the control groups. The concordance between rA2-ELISA and rA2 latex compared with DAT as a gold standard serological test for VL were found 73.7% and 77.5%, respectively.

**Conclusion:**

A good degree of agreement was found between rA2-ELISA and DAT (73.7%). rA2-ELISA could detect more seropositive serum samples than rA2-LAT and it may be recommended as an alternative tool for the diagnosis of CVL.

## Introduction

Visceral leishmaniasis (VL), or Kala-Azar, is a zoonotic disease due to *L. Infantum* transmitted by female sand flies and caused by *Leishmania* species which is fatal if left untreated (1). Domestic dogs (*Canis familiaris*) are the principal reservoir host of Mediterranean type of VL (2, 3).

Clinical signs of infective dogs are classified into symptomatic and asymptomatic. In severe forms (symptomatic), the dogs represent cachexia and weakness, baldness, loss of appetite, weight loss, keratoconjunctivitis, and death (2). Asymptomatic dogs are source of *L. infantum* infection as well as symptomatic dogs (3, 4).

The risk of parasite transmission from dogs to sand flies and humans is increased by close contact between dogs and human population (5). Diagnosis of canine visceral leishmaniasis (CVL) is difficult because dogs present variety of clinical signs and many dogs are asymptomatic. Different procedures have been used for the diagnosis of VL including parasitological methods based on aspirates or biopsy of visceral tissues (liver, bone marrow, and spleen). Parasitological diagnosis for finding leishmanial amastigotes in stained smears remains the gold standard in the diagnosis of leishmaniasis; however, this method is characterized as an invasive assay. The in vitro cultivation of parasites from any of the above samples requires sophisticated laboratory facilities. Different serological methods are also used widely for diagnosis of VL varying in sensitivity and specificity such as indirect fluorescent antibody (IFA) (6), direct agglutination test (DAT) (7) and ELISA (8). The rapid immunochromatographic dipstick test is a qualitative test able to detect anti-*Leishmania* circulating antibodies by the leishmanial recombinant antigen, such as rK39 (9, 10), rKE16 (11) and is adapted for use under field conditions.

A difficulty of the DAT is the relatively long incubation time and the need for serial dilutions of blood or serum. The ELISA and IFA test require technological expertise and specialized laboratory equipment (12). Rapid and early detection of human and dogs *L. infantum* infection is important for surveillance and control programs and a fast, sensitive and non-invasive tool is highly necessary, as it will enable quick treatment and therefore decrease the mortality rate of human VL (13). Immunological methods based on the agglutination of latex particles widely used in biology for the detection of small quantities of an antibody or antigen in fluid sample and it is a simple method and the results obtained in a short time (14).

Recombinant antigen-based LAT is a suitable technique for the examination of a large number of sera. This test is extremely simple and rapid and can be performed in condition where facilities or resources to perform more complicated tests are not available. For this purpose, we evaluated the rA2-LAT and rA2-ELISA assays for detecting the anti-visceral leishmaniasis antibody in symptomatic and asymptomatic dog’s sera from Meshkin-Shahr North West of Iran and comparing the efficacy of LAT and ELISA test for diagnosis of VL with DAT.

## Materials and Methods

### Development of recombinant A2 antigen

Under the A2 gene sequences available in Gene Bank Databases with Accession number NO: AY255808, a part of the A2 gene sequences which contain immune dominant sequences and less number of repetitive sequences was selected. Therefore, we predict a 6 histidin in the C terminal, synthesized by Sinaclon Company, Iran. The sequences of A2 gene are as follow:

CAGGAAACAGCTATGACCATGATTAC-GCCAAGCTGCCCTTCCATGGGGTCCCTG-CAGGACTCAGAAGTCAATCAAGAA-GCTAAGCCAGAGGTCAA-GCCAGAAGTCAAGCCTGA-GACTCACATCAATTTAAAGGTGTCCGATGGATCTTCAGAGATCTTCTTCAA-GATCAAAAAGACCACTCCTTTAA-GAAGGCTGATGGAA-GCGTTCGCTAAAAGACAGGG-TAAGGAAATGGACTCCTTAACGTTCTT-GTACGACGGTATTGAAATTCAA-GCTGATCAGACCCCTGAAGATTT-GGACATGGAGGATAACGA-TATTATTGAGGCTCAC-CGCGAACAGATTGGAGGTGAACCG-CACAAAGCGGCAGTGGATGTT-GGCCCGCTGAGCG-TAGATGTGGGTCCGCTGTCCGTT-GGTCCTCAGTCCGTGGGTCCGCTGTCCG-TAGGCCCGCAAAGCGTAGGTCCAC-TGTCCGTCGATGTCGGCCCTCTGAGCG-TAGGCCCGCAGAGCCATCACCAC-CATCATCAC-TAAGGATCCGAATTCGAGCTCAAGCTT-GCGGCCGCCTCGAG.

Briefly, the A2 Gene (pEASY–A2) was transformed to Top10 *E. coli* competent cell by thermal shock (30 min on ice, 90 sec thermal baths at 42 °C). The recombinant plasmid (pEASY-A2) and expression vector (pET22) were digested by *E*coRI and *N*coI enzymes. The products of digestion were analyzed by electrophoresis on 1% agarose gel. In order to express recombinant protein (pET-A2), *E-coli* BL21/DE3 cells were used as host cell. The expression of A2 was induced at OD 600 of 0.6 by adding of 1mM of Isopropyl-β-D-Thiogalactopyranoside (IPTG) and incubation at 37 °C. The A2 expression was analyzed by 12% SDS-Polyacrylamide Gel Electrophoresis (SDS-PAGE) (15). The purification of rA2 proteins applied via Ni-NTA affinity chromatography. For purification, 200 ml culture media were collected, centrifuged and re-suspended in 10 ml of binding buffer (50 mM Tris-Cl, 5 mM imidazole, 500 mM NaCl). After sonication of the cells suspension for 7 min, the supernatant was applied to the column. Then the column was washed step-wise with 25, 20, 15 and 10 ml of binding buffer containing 20, 40, 60 and 80 mM of imidazole. Finally, the adsorbed protein was eluted with binding buffer containing 400 mM imidazole (16). The antigenicity of A2 protein was surveyed in western blotting using both pooled dog sera and C9 anti-A2 monoclonal antibody.

### Sampling

This study was conducted over a period of 2 yr from 2011 to 2013 in the Meshkin-Shahr district in the northwest part of Iran, where visceral leishmaniasis is endemic (17,18).

Overall, 350 dog sera were collected and screened from both male and female domestic dogs of various breeds and ages. The animals were tested after obtaining ethical permission from the dog owners. An expert veterinarian was clinically examined the dogs. Dogs blood samples (2.5 ml) were collected by venipuncture, centrifuged at 800 g for 5–10 min, and the sera were separated and stored at −20 °C until tested. Each serum sample was tested for anti-leishmanial antibodies by using the standard DAT in the Leishmaniasis Laboratory of the School of Public Health at Tehran University of Medical Sciences (12). Suspected domestic dogs in addition to at least two classical signs (lymphadenopathy, alopecia, skin ulceration, anorexia, dermatitis, epistaxis, keratoconjunctivitis, onychogryphosis, lameness, and weight loss) with sera titer for *Leishmania* antibody ≥ 1:320 by DAT was considered as symptomatic dogs. Positive samples with titer 1:80 and 1:160 by DAT with no signs and negative samples were considered to correspond to asymptomatic and uninfected dogs, respectively.

### Direct Agglutination Test (DAT):

The *L. infantum* antigens for this study were prepared in the protozoology unit of the School of Public Health at the Tehran University of Medical Sciences. Briefly, the procedure for preparing the DAT antigen was mass production of promastigotes of *L. infantum* in RPMI-1640 medium supplemented with fetal bovine serum (FBS), trypsinization of the parasites, staining with Coomassie Brilliant blue and fixing with formaldehyde. Serial dilutions of serum were prepared to start at a dilution of 1:10 to a maximum dilution of 1:20480. Antigen was added to each microplate wells. In position of a positive test, the wells represented a cloudy condition, otherwise during a negative result; the point is deposited at the bottom of well. All canine serum samples were tested by DAT according to this method (19).

### Latex agglutination test (LAT)

Latex beads, 0.8 μm (Sigma, USA) were sensitized with rA2 antigen according to the methods (17) with some modifications. Latex beads were added to 0.1 M PBS (pH 7.6) to obtain a dilution of 1:10. The suspension was washed twice by centrifugation at 12000 rpm/4 °C for 5 min in 0.1 M PBS. Finally, the latex beads were made into a 10% suspension later mixed with an equal volume of rA2 antigen. The mixture was incubated at 37 °C for 2 h with constant shaking at 40–50 shakes/min for adsorption. At the end of this procedure, 5 mg/ml (w/v) bovine serum albumin was added to the beads sensitized with the rA2 antigen and stored at 4 °C until use. An aliquot of 10 μl serum in 0.1 M PBS, pH 7.6, was mixed with 10 μl of latex antigen, and the solution was shaken at room temperature for 3–5 min. After 5 min, the slide was visually inspected for clearly visible agglutination of latex particles, which would occur if the serum sample contained any anti-rA2 antibodies.

### ELISA

Protein concentrations were calculated by the Bradford assay (20) with bovine albumin as the standard. ELISA procedure was optimized with regard to antigen concentration and conjugate dilution. Positive-control sera from dogs with parasitological infection; DAT positive (≥1:320) and negative-control sera were included in assay. Sera samples from dogs were analyzed by standard micro-ELISA (21) to detect antigen-specific antibodies. Recombinant A2 proteins were diluted in 0.1 M carbonate-bicarbonate buffer (pH 9.6) containing 0.02% NaN_3_. One hundred microliters per well were used to coat 96-well microtiter plates (Nunc, Roskilde, Denmark) overnight at 4 °C. Free binding sites were blocked with a 1% BSA solution for 2 h at 37 °C. After three washes with PBS-Tween20 (PBS-T) at 0.05%, the plates were incubated with 100μL of canine sera diluted 1:100 in phosphate buffered saline (PBS-T) for 1 h at 37 °C. The plates were then washed three times with PBS-T and incubated with 1:20000 anti-canine IgG antibody (Sigma, USA) conjugated with horseradish peroxidase. The reaction was stopped by the addition of 100μL of a 1M solution of HCl per well. The optical density (OD) was measured at 450 nm in an ELISA microplate spectrophotometer (Molecular Devices, BIO-Tek, USA). The lower limit of positivity (cut-off) was determined by the OD mean of 20 negative canine control sera plus two standard deviations.

### Relative sensitivity, specificity, and Concordance

Relative sensitivity, specificity of test for detection of anti-leishmanial antibodies in dog sera were determined and the test parameters of the rA2-ELISA and the rA2-LAT were compared to the corresponding parameters of DAT, considered the gold standard for serological diagnosis of CVL.

*Sensitivity* =TP/TP+FN (TP: True Positive, FN: False Negative)
*Specificity* =TN/TN+FP (TN: True Negative, FP: False Positive)
Concordance=TP+TN/Total samples

## Results

**Fig. 1:**
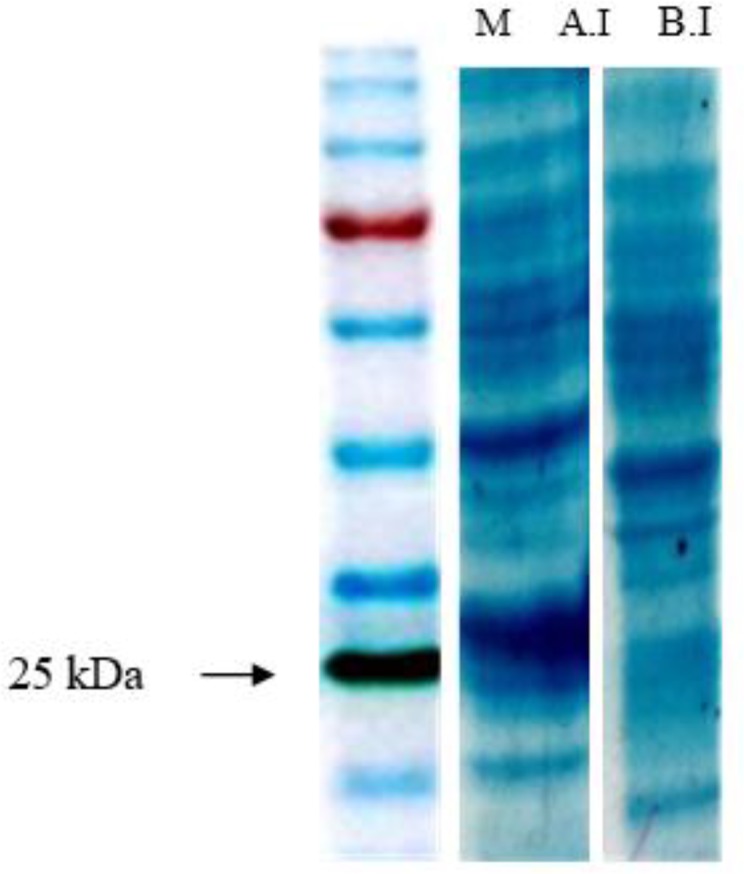
SDS-PAGE analysis of expression of rA2 in *E. coli* SDS-PAGE analysis showed the expression of an approximately 25 *kDa* protein in induced bacteria (AI) which was absent in uninduced bacteria (BI).M: Protein molecular weight marker

**Fig. 2:**
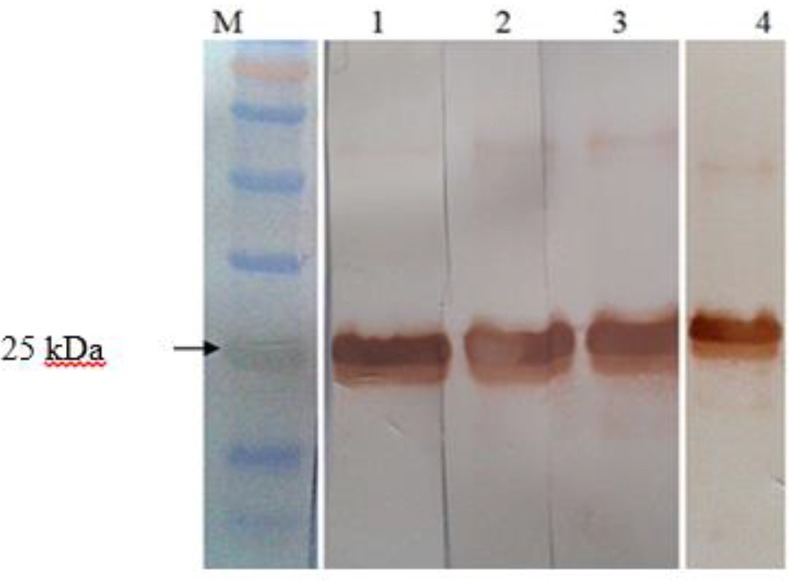
Western blot analysis of purified rA2 protein among different dilution of anti-A2 C9-monoclonal antibody: 1:1/70, 2:1/50, 3:1/40, 4: primary antibody pooled dog serum 1/100. M: protein ladder 11-160 kDa

SDS-PAGE analysis of induced bacteria showed expression of rA2 protein, which was absent in un--induced bacteria. The antigenicity of A2 protein was surveyed in western blotting using both pooled dog sera and C9, an anti-A2 monoclonal antibody (Fig. 1, 2).

**Fig. 3:**
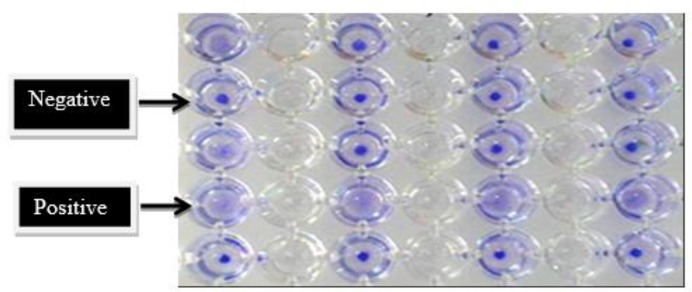
DAT test

In position of a positive test by DAT, the wells represented a cloudy condition, otherwise during a negative result; the point is deposited at the bottom of well (Fig. 3).

**Fig. 4:**
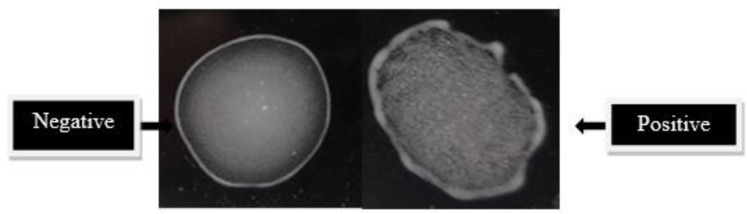
Latex Agglutination Test

A rapid and simple LAT was developed using rA2 antigen. While the serum was diluted 1/2 – 1/32 with PBS, strong agglutination with positive serum was observed at dilution of 1/32 and it is suggested for serum dilution. Positive results were read on a +1 to +4 scales depending on the extent of agglutination and if no agglutination was observed within 5 min the test was negative (Fig. 4).

Among these samples, 29 sera were positive by latex and the relative sensitivity, specificity and concordance of the test were based on DAT test. In symptomatic dogs the relative sensitivity and specificity of rA2-LAT were 25.71% (95% CI 16.01%–37.56%) and 95.53% (95% CI 92.14%–97.75%) respectively and concordance of 80.1% (95% CI,75.2%–84.3%) while in asymptomatic dogs were 5% (95% CI 2.25%–7.86%) and 74.29% (95% CI 62.44%–83.99%) and concordance of 19.9% (95% CI, 15.7%–24.8%) with DAT test.

ELISA procedure was optimized with regard to antigen concentrations and the serum dilution optimal antigen concentrations were 0.1μg/ml. When we tested sera by rA2-ELISA, this test could detect anti leishmainial antibody in 96 (27.4%) of sera and the relative sensitivity and specificity of rA2-ELISA in symptomatic dogs were 62.7% (95% CI 50.01% to 74.20%) and 80.92% (95% CI 75.85% to 85.33%) while in asymptomatic infected dogs, were 17.6% (95% CI 13.09% to 22.90%) and 48% (95% CI 37.90% to 58.22%), respectively with ≥1/80 cut-off titer of DAT-confirmed cases (Table 1 & 2).

**Table 1:** Comparison of the performances of DAT *, rA2-ELISA and rA2-LAT for diagnosis of CVL

***Type of technique***	***Positive***	***Negative***	***Total***
DAT	97	253	350
ELISA	96	254	350
LAT	29	287	316

DAT* cut-off ≥1/320

**Table 2:** Seropositivity rates of rA2-ELISA and rA2-LAT compared to DAT among symptomatic and asymptomatic dogs

***Type of technique***	***Symptomatic^1^***			***Asymptomatic^2^***			***Total***
rA2-ELISA	62.7	37.3	67	16.1	83.9	230	297
rA2-LAT	27.3	72.7	55	5 95		222	277

DAT^1^ cut-off ≥1/320 with at least two classical signs

DAT^2^ cut-off = 1:80, 1:160

P^*^: Positive, N**: Negative

In symptomatic and asymptomatic dogs the concordance of rA2-ELISA with DAT as a gold standard test were 77.4% (95% CI,72.7%–81.7%) and 26.2% (95% CI,21.7%–31.2%), respectively.

## Discussion

In the present study, a rapid rA2- LAT was developed for the first time to detect visceral antibodies from dog samples. The sensitized beads could detect the antibodies against the canine VL (CVL). The results obtained with latex using samples collected from symptomatic and asymptomatic dogs indicated that the sensitivity of the test was 27.27%; however, its specificity was 94.64% in symptomatic dogs. LAT as a useful assay in difficult field conditions because it is simple, easy to perform, inexpensive, does not require any electric equipment, and is read visually for routine screening of VL in endemic areas where laboratory facilities are limited. The sensitivity of the test was low and must be developed in the future works.

This is comparable with the only recombinant antigen used for detection of anti-leishmanial antibody by the rk39 latex test. This antigen is generally used in LAT at a concentration of 1 mg/ml in glycine-buffered saline. The sensitivity of the latex test among parasitologically proven index cases was only 80%, which was due to a lower power of antibody detection by this test (21). Fraction of A2 antigen was used (2013) for LAT, who indicated the A2-LAT of canine sera from DAT-confirmed cases yielded a sensitivity of 95.2% and specificity of 100% (17).

In previous publications, rA2 was used in other assays leading different results. Recombinant A2-ELISA was another diagnostic test (2015) used for detection of rA2 antibody in dog’s sera. In their study, the sensitivity and specificity of this test were 52.58% and 82.21% respectively in symptomatic dogs (11). An ELISA technique was used based on recombinant leishmanial antigens (rA2) for serodiagnosis of symptomatic and asymptomatic *L. infantum* visceral infections in dogs. Sensitivity of rA2-ELISA was 76% in symptomatic dogs and 89% in asymptomatic dogs but the rA2 specificity was 96% (22). In contrast with their research, the sensitivity of the rA2-ELISA in asymptomatic dogs were low which may explain low concentration of anti-*Leishmania* antibodies in asymptomatic dogs. Due to the low sensitivity to detect asymptomatic infected dogs, it is not indicated for large-scale in epidemiological studies we suggest more research in future.

Serologic test performance depends on many factors such as infection status and the type of diagnostic antigen. There might be some differences in immune responses of CVL to VL and explain this disagreement but in serological tests, the problems due to sensitivity and specificity also arise (23).

Although there are several antigens represented by both amastigotes and promastigotes stages of *Leishmania* parasites (5, 23), however the most expression of rA2 proteins has only observed in the amastigotes one (24, 25).

## Conclusion

Agreement of the rA2-LAT and rA2-ELISA when compared with the DAT as a gold standard were 77.4% and 26.2% respectively for rA2-ELISA and 80.1% and 19.9% for rA2-LAT in symptomatic and asymptomatic dogs respectively. Both LAT and ELISA test may be applied for detection of Anti-*Leishmania* antibodies in symptomatic dogs and but rA2-ELISA could detect more seropositive serum samples than rA2-LAT in symptomatic and asymptomatic dogs. However, more study is needed to develop the test by using other concentrations and sizes of latex beads in order to reduce the rate of false-negative reactions.
